# Depression and stress amongst undergraduate medical students

**DOI:** 10.1186/s12909-015-0425-z

**Published:** 2015-08-27

**Authors:** Allison B. Ludwig, William Burton, Jacqueline Weingarten, Felise Milan, Daniel C. Myers, Benjamin Kligler

**Affiliations:** 1 Albert Einstein College of Medicine, 1300 Morris Park Ave, Bronx, NY 10461 USA; 2 Montefiore Medical Center, 111 E. 210th Street, Rosenthal Pavilion, Room 4, Bronx, NY 10467 USA; 3 Albert Einstein College of Medicine, Clinical Skills Center, 1300 Morris Park Avenue, Bronx, NY 10461 USA; 4 Beth Israel Medical Center, Continuum Center for Health and Healing, 245 Fifth Avenue, New York, NY 10016 USA

**Keywords:** Wellness, Depression, Burnout, Stress, Curriculum

## Abstract

**Background:**

The demands placed on medical trainees pose a challenge to personal wellbeing, leading to burnout and erosion of empathy. However, it is unclear at what point in medical education this decline begins. Although many schools have begun to design and implement wellness programs for their students, the medical education community’s experience in evaluating their impact is limited.

**Methods:**

The authors designed a wellness needs assessment of all medical students at the Albert Einstein College of Medicine in order to assess students’ health behaviors, stress and depressive symptoms. The online survey was administered to all medical students from the classes of 2014 and 2015 at the beginning of their first year of medical school and again at the end of their third year. Chi-square and T-tests were run comparing the survey responses of the two classes.

**Results:**

There was a significant increase in perceived stress from an average of 5.51 in the first year to 6.49 in the third year (*p* = .0001). The number of students at risk for depression, defined as a CES-D score greater than 16, was 94 (28.4 %) in the first year and 131 (39.0 %) in their third year (*p* = .004).

**Conclusions:**

This study demonstrates a significant increase in the proportion of students at risk for depression in their third year as compared to the first year as well as an increase in perceived stress. In response to these findings, the authors took a multi-disciplinary approach in the development of a comprehensive program to address student wellness, including efforts to address issues specific to the clinical clerkships. This program is unique in that its design, inception and ongoing evaluation have taken the needs of an entire medical school class into account.

## Background

It is well known that the demands and pressure of medical school and residency pose a tremendous challenge to personal wellness for physicians in training, leading to high rates of anxiety, depression, burnout and personal distress [1]. Studies have documented that increased personal wellbeing is associated with enhanced empathy among both medical students [2] and residents [3]. Therefore, the ability of physicians in training and practice to function as humane, humanistic doctors is compromised by these challenges to the physician’s own health and sense of balance [4].

To some degree, the medical education establishment has called for schools to rise to the challenge of this new understanding of the risks of medical training for our trainees. The Association of American Medical Colleges [5], Institute of Medicine [6], and the Liaison Committee on Medical Education (LCME) have promoted the need to address physician wellbeing in medical education [7], and wellbeing is now being formally evaluated as part of the widely referenced Graduation Questionnaire (GQ). As a result, many medical schools have designed and implemented programs to promote trainees’ wellness [8]; however the medical education community’s experience in evaluating the impact of these interventions is still limited.

In this article, we will describe how a class-wide needs assessment at the Albert Einstein College of Medicine informed the development of the WellMed program, a comprehensive approach to student wellness. Similar medical school programs have aimed to impact the wellness attitudes and behaviors of the entire student body, but specific interventions have often been limited to subgroups of medical students. Our program, from its design phase to implementation and evaluation, has incorporated the feedback from the entire student body and created wellness activities that are part of the required curriculum for all students.

## Methods

### Needs assessment

The first phase in the assessment was a qualitative analysis of the reflective essays of an entire third year class. The major themes which emerged from this research were: 1) managing one’s own health choices; 2) the burden of becoming a role model; 3) the impact of newly acquired medical knowledge and experience of patients’ illness on the students’ perspective on their own health; and 4) the relationship between wellness and professional identity [9].

The second phase was an online wellness survey consisting of 50 questions that included previously validated measures of depression (Center for Epidemiologic Studies Depression scale or CES-D), [10], stress (Perceived Stress Scale or PSS) [11] and sleep habits and substance use (National Health Information Survey or NHIS) [12], and a standard measure of diet and exercise (the Weight, Activity, Variety and Excess tool or WAVE [13]), Although we did not undertake validating the entire 50 question instrument as a whole, we did choose previously validated measures where they were available (depression, stress, sleep and substance use), balancing the “research” need to create a valid survey instrument with the “curriculum” need to have this assignment be reasonable in the amount of time required for students. In the nutrition and exercise domain, the validated measures available were too lengthy to be practical for this required survey, so we used the WAVE, a widely used clinical survey tool. Students were required to complete this survey during the first two months of first year as part of the Introduction to Clinical Medicine course. Individual survey results were kept confidential and without identifiers, but aggregate class results were available for analysis. Students were then required to repeat the same survey near the end of third year as part of the Patients, Doctors and Communities course. The results of our baseline sample of students from the Classes of 2014 and 2015 during their first three years of medical school are presented here.

Since there were no individual identifiers, this study was granted exemption by the Einstein Institutional Review Board.

### Statistical analysis

Chi-square and T-tests were run comparing the survey responses of the students in their first and third years. Because no identifiers were included in the surveys, the groups were treated as being independent rather than paired. We performed all data analyses using SAS version 9.3 (SAS Institute, Cary, North Carolina). The CES-D scores were dichotomized to identify those at high risk of depression, defined by a score of 16 or higher (out of a possible score of 0–60) [14].

## Results

We analyzed the combined results for the Classes of 2014 and 2015. Since this was a required assignment in both the Introduction to Clinical Medicine Course and the Patients Doctors and Communities Course, the total number of respondents to the first year survey was 332 and the total number of respondents to the third year survey was 336 with a response rate of 100 % on both surveys.

**Fig. 1:**
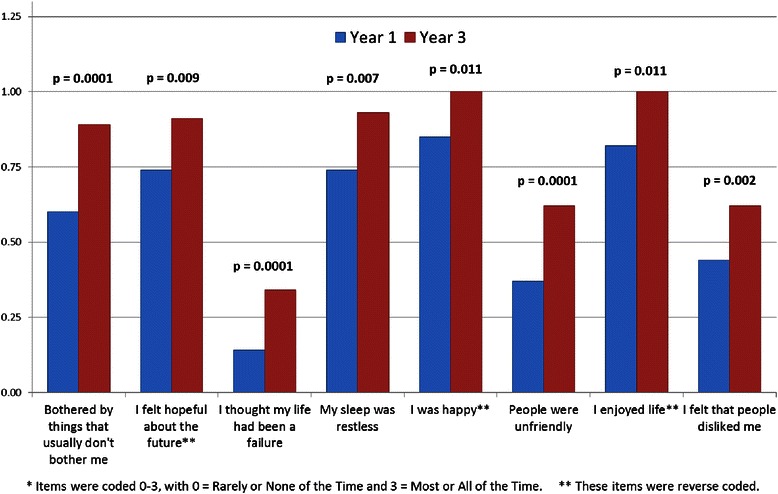
Comparison of 1st and 3rd year students on depressions items*

The number of students with a CES-D score greater than 16 in the first year was 94 (28.4 %) and 131 (39.0 %) in their third year (*p* = .004). As shown in Fig. 1, there were 8 individual items on the CES-D in which students in their third year scored higher (i.e., showed greater evidence of depression) than students in their first year.

Regarding the Perceived Stress Scale, the overall sum of the four questions went from an average of 5.51 in the first year to 6.49 in the third year (*p* = .0001). Additionally, all four individual items achieved statistical significance in regards to increased perceived stress between MS-1 and MS-3 year, specifically “I felt unable to control the important things in my life;” “I felt confident in my ability to handle personal problems;” “I felt things were going my way;” and “I felt difficulties were piling up so high I could not overcome them.”

## Discussion

To address the above findings, and the documented risk of increased depression during the course of medical school, we created WellMed, a coordinated wellness program that incorporates multiple dimensions of wellness into one cohesive program. Inspired by similar programs at Vanderbilt and elsewhere [8], the components included in the program are physical health; mental health; spiritual wellness; physical fitness; nutrition; intellectual wellness; social wellness; and financial wellness. Under the leadership of the Assistant Dean for Student Affairs, a task force was organized to expand and effectively “market” the program on campus. The group included additional faculty members, the Director of Student Activities and the Director of Student Affairs, the Director of Fitness Services, Director of Food Services and representative students from all four years of the medical school curriculum. This program was not meant to replace the existing services available for students, but rather to complement and enhance the existing infrastructure. For example, prior to the program’s implementation, students in psychological distress received referrals for counseling through the Office of Academic Support and Counseling. While this is still a way for students to receive acute counseling and referrals, students can also access psychiatric services directly through a partnership developed between WellMed and the Department of Psychiatry that allows students direct access to expedited psychiatric services.

Since the introduction of the program in the 2013–2014 academic year there have been multiple WellMed sponsored events around campus. Based on our needs assessment findings, a mindfulness training program has been integrated into the established and required “transition to third year” program which is administered just before the students begin their first clerkship. Additionally, there has been an emphasis on teaching stress reduction strategies to the Einstein Peer Mentors, a group of second, third and fourth year students dedicated to supporting members of the community throughout their tenure at Einstein. Sessions have included simple massage techniques and meditation.

### Future directions

In the coming year we plan to incorporate mindfulness interventions into the programming in ways to reach the entire class. Mindfulness interventions have been shown to lead to reduction in depression and anxiety in first and second year medical students [15, 16], but their impact on the clerkship years has not been examined. Our goal in this effort will be to develop a model for mindfulness training compact enough to be offered to the entire class as part of the required curriculum, and yet effective enough to make a meaningful difference. The intervention will start during second year and then continue into and through the third year and will be based on models successfully developed and implemented for smaller groups of students elsewhere [17]. Although we recognize that specific mental health and counseling interventions will still be necessary for many students to address the increased stress and depression documented here, we hope to reduce the proportion of the class in need of these interventions through this class-wide strategy.

We will continue to administer the wellness survey as a curricular element to the first and third year classes. We plan to compare the above baseline results to subsequent classes who have been offered the WellMed program since matriculating at Einstein. We will supplement this quantitative data with a qualitative inquiry using focus groups with third year students from the classes of 2017–2018. The purpose of the qualitative inquiry will be to glean a more nuanced view of the challenges to student wellness, to solicit feedback on the elements of the program that students felt were helpful and to gain insight into future directions.

### Limitations

Although our comprehensive needs assessment and evaluation strategy for our wellness program has distinct advantages, some limitations must be noted. First, the fact that the wellness survey is required may affect the honesty of reporting due to students’ concerns regarding whether their answers are in fact entirely confidential. Second, to protect this anonymity, we were unable to include specific identifiers to track the impact of our program on individual students. Therefore, we can only evaluate the data for the class as a whole, possibly leading to an underestimate of the actual changes that happen during medical school for selected students more at risk and of the potential impact of our program on those same students. Finally, as multiple components to the program may be implemented over the same time period, it is difficult to discern the impact of any one intervention on the overall wellbeing of the class.

## Conclusion

There is a growing emphasis on wellness in medical education nationally and internationally. However, many of these innovative new programs target small selected groups of students [18–20], and their relevance to the entire population of medical students is unclear. Our needs assessment suggests an urgent need for wellness programs. Given the challenges in identifying the students most at risk, programs that address the entire student body are needed. Our strategy with the WellMed program demonstrates the type of comprehensive approach we feel will be most effective: the planning of the program was based on longitudinal survey data using validated quantitative measures; the focus of the program is expansive, targeting all four classes and eight domains of wellness; and the evaluation strategy uses survey data required as part of two specific classes, allowing us to describe the impact of our program on the entire student body.

Ultimately, the goal of the WellMed program is to create a culture where taking care of oneself is felt to be as valuable as caring for others. A core component of our strategy to reach as many students as possible is a conscious effort to use the WellMed “brand” with the goal of making students aware on a daily basis that the school considers wellness a critical part of their education as professionals and not simply a sideline activity.
